# Supplementary material for: Entrapment of N-Hydroxyphthalimide Carbon Dots in Different Topical Gel Formulations: New Composites with Anticancer Activity
Source: Pharmaceutics. 2019 Jul 1;11(7):303. doi: 10.3390/pharmaceutics11070303 (PMC6680570; doi:10.3390/pharmaceutics11070303)
Supplement: Supplementary file 1 [file pharmaceutics-11-00303-s001.pdf]

pharmaceutics

Supplementary Materials: Entrapment of *N*-Hydroxyphthalimide Carbon Dots in Different Topical Gel Formulations: New Composites with Anticancer Activity

Corina-Lenuta Savin, Crina Tiron, Eugen Carasevici, Corneliu S. Stan, Sorin Alexandru Ibanescu, Bogdan C. Simionescu and Catalina A. Peptu

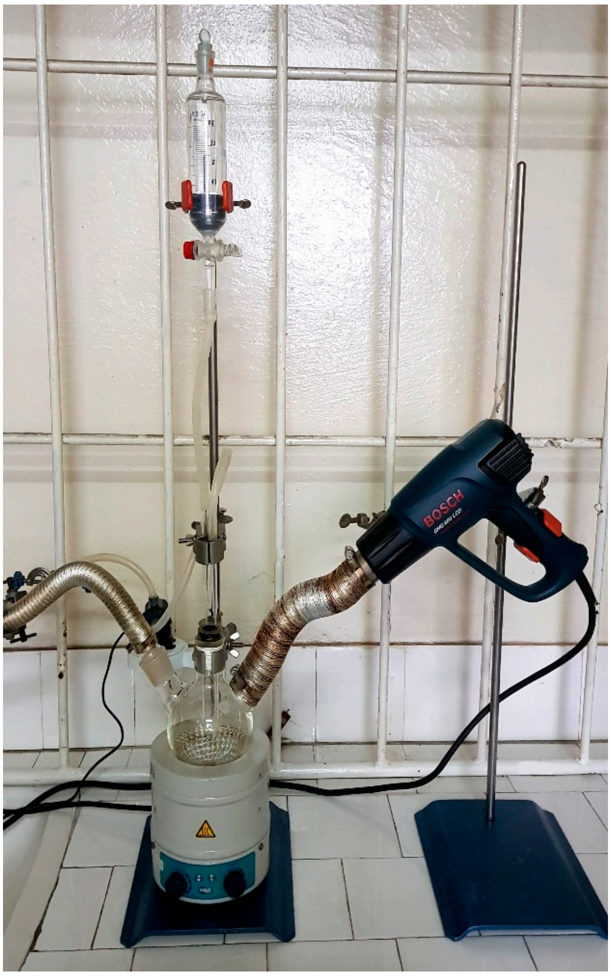

Figure S1. Experimental setup used to prepare carbon dots.

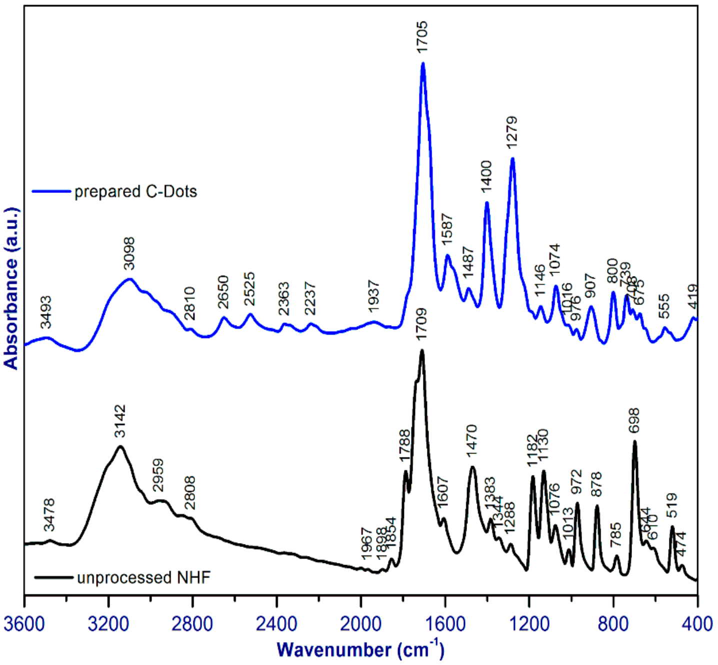

Description	Recorded peak (cm^{-1})	
	NHF	C-Dots
OH stretch	3142	3098
C=C stretch	2959, 2806	2810, 2650
C-C stretch	1854	-
C=O sym. stretch	1788	-
C=O asym. stretch	1709	1705
C-H def.	1607	1587
N-O stretch	1470	1487
C-C stretch	1383, 1288	1400, 1279
N-OH bend<br>C-N stretch	1182	1146
succinic ring torsion	972	-
succinic ring	698	-
aromatic ring bending	519	555

Figure S2. FT-IR spectra for CD-NHF.

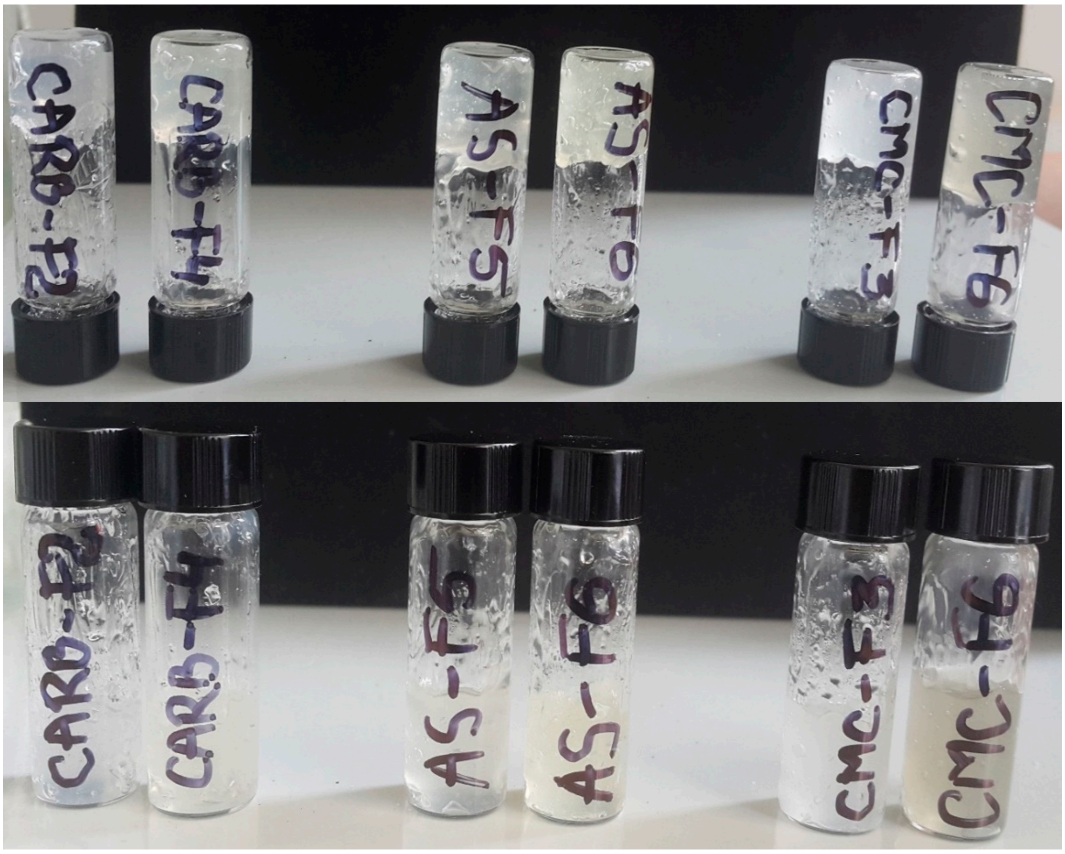

(a)

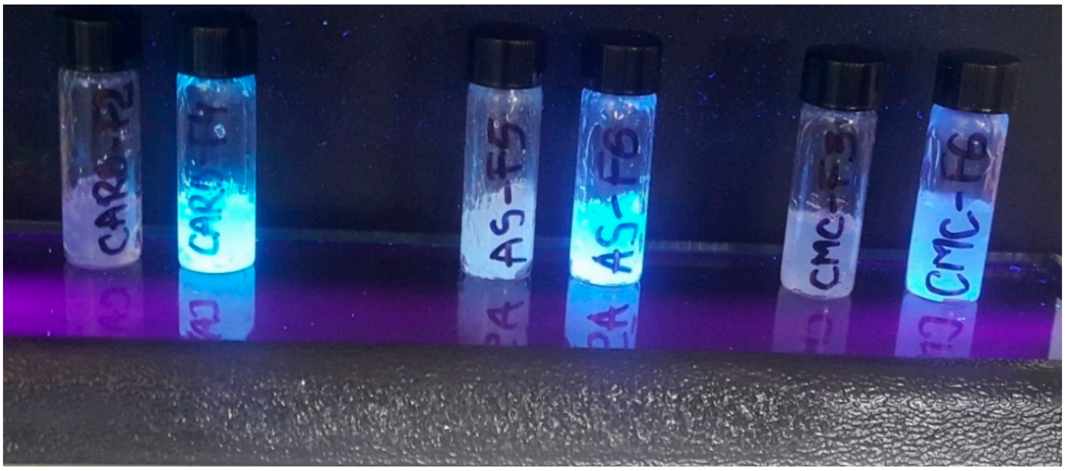

(**b**)

Figure S3. Photos of gels without and with CD-NHF under (a) white and (b) UV light.

From Figure S4 can be observed the acquired microscopic pictures displaying separate staining channels of color (green for cell viability, blue as nuclear counterstaining) and merged images.

Supplementary Figure S5 displays the analytical segmentation procedure for a typical spheroid image. Fluorescent pictures were acquired using a conventional, standard Zeiss Axio Observer Z1 Microscope in a single focal plane.

Under these circumstances, the software quantification of the viability figure for a target spheroid cumulates fluorescence from cells situated on the focal plane (where microscope capture maximum of fluorescent intensities) and from surrounding planes (resulting in more-blurred signals). While unavailable here, a confocal microscope would provide better accurate images of the interior of 3D spheroids.

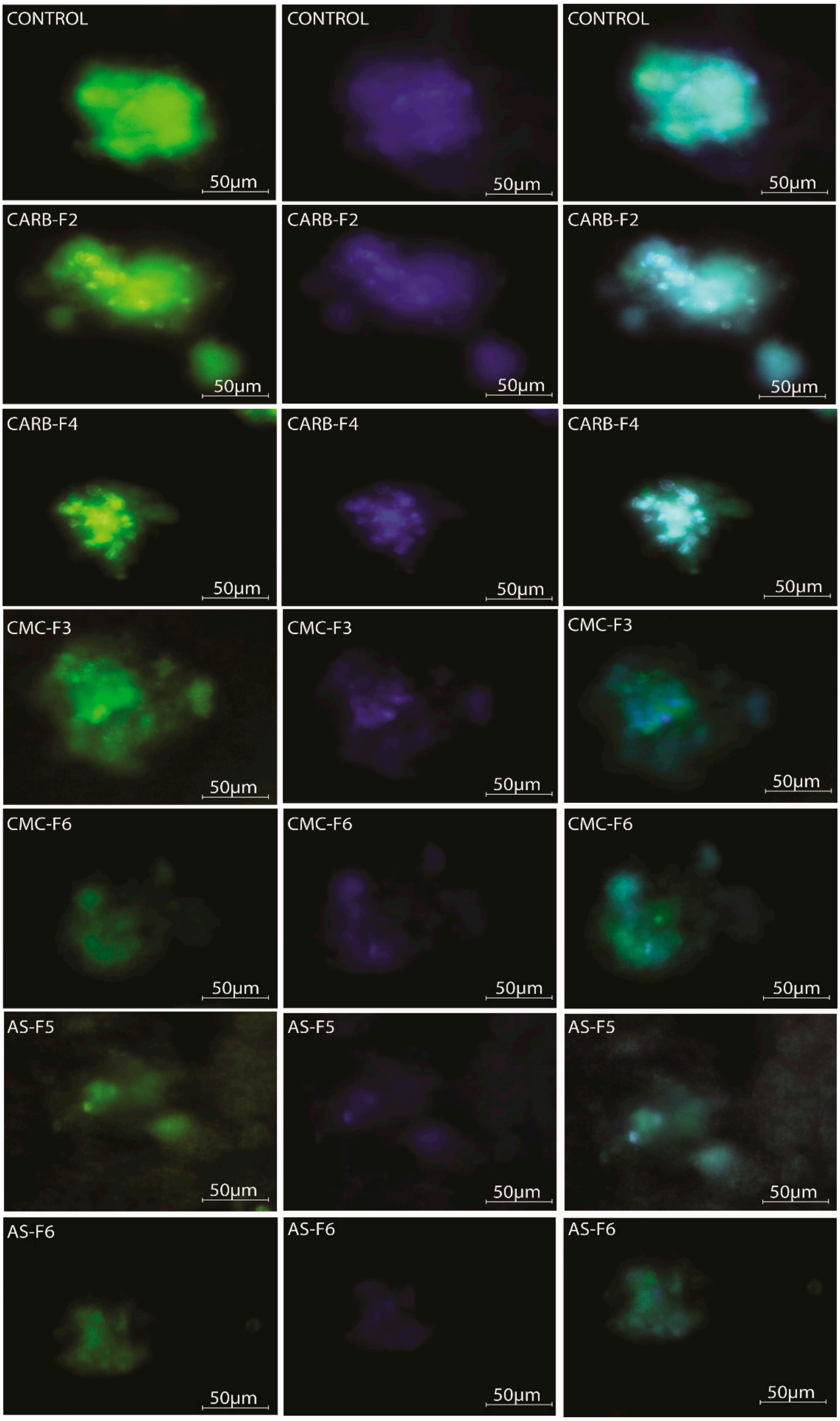

Figure S4. Spheroid fluorescent staining using green/FITC (left column) for live cells, blue/NucBlue for nuclei (middle column), and merged signals (right column) from 3D human melanoma cell cultures.

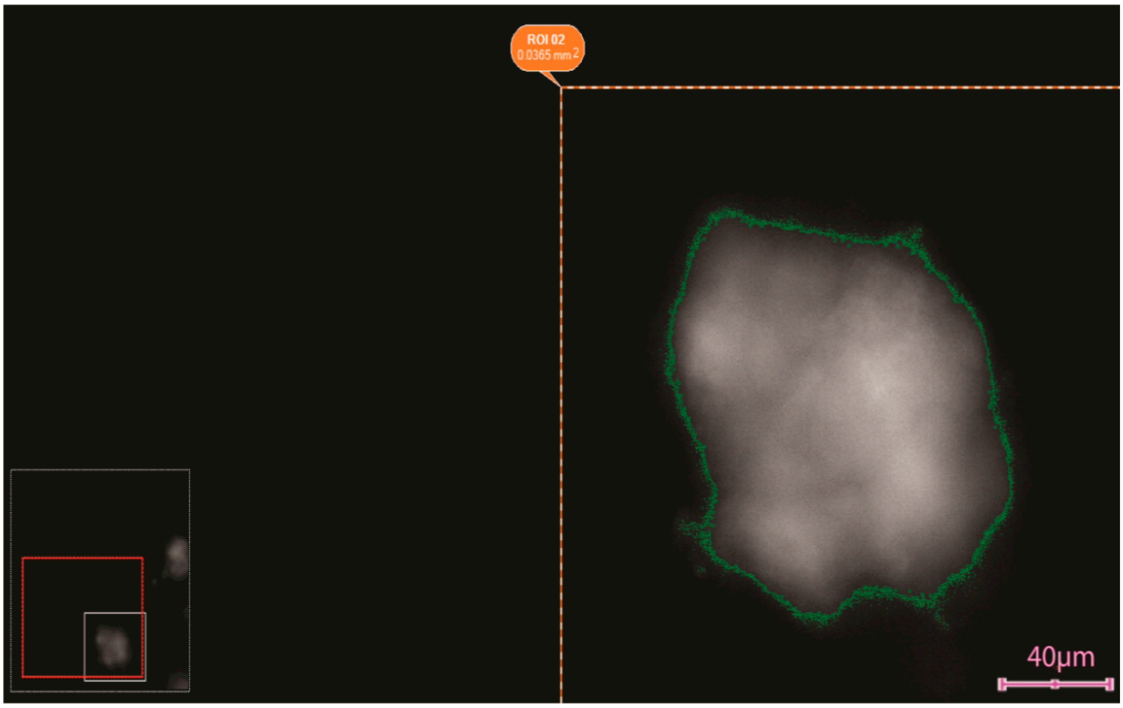

Figure S5. The analytical segmentation procedure for a typical spheroid image.

Table S1. Fluorescence results obtained for CD-NHF-loaded gels at different excitation wavelengths: 370–410 nm.

Sample code	Excitation (nm)	Emission peaks (nm)	Intensity (Counts)
CD-NHF	370	424	1.27401×10^6
CARB-F4	370	425	1.25371×10^6
AS-F6	370	425	1.12806×10^6
CMC-F6	370	425	1.12806×10^6
CD-NHF	390	441	938,927
CARB-F4	390	441	956,280
AS-F6	390	439	741,741
CMC-F6	390	439	741,741
CD-NHF	410	476	622,313
CARB-F4	410	473	566,354
AS-F6	410	474	392,623
CMC-F6	410	474	392,623
